# An Instrument for Determination of Energy Oxygen and BOD_5_

**DOI:** 10.6028/jres.093.057

**Published:** 1988-06-01

**Authors:** Leonard L. Ciaccio, Klaus Hameyer

**Affiliations:** Ramapo College, Mahwah, NJ; Chemical Abstracts Service, Columbus, OH

“The biochemical oxygen demand (BOD) determination is an empirical test in which standardized laboratory procedures are used to determine the relative oxygen requirement of waste waters, effluents and polluted waters [1].” This method has several well-known drawbacks, i.e., 5-day duration of the test, lack of correspondence of the BOD bottle to the biological system of the receiving water body, and the nonquantitative nature of the BOD test [2]. Each of these factors seriously compromises the present testing procedure. The length of the analysis time, which is generally 5 days, makes the test practically useless [2,3] in real-time control of pollution, and the prescribed condition of carrying out the test at a constant temperature of 20 °C is an inconvenience.

The objective of this investigation was to develop an instrument which would measure biological oxygen demand by some parameter which was meaningfully related to the oxygen depleting activity of waste waters, precise, and of short measurement duration (1 hour or less). The variable chosen was energy oxygen, a thermodynamic value related to the substrate free energy of oxidation in cell synthesis [2–8].

Two factors, energy oxygen and endogenous oxygen, comprise ultimate biochemical oxygen demand [2,3,4]. Five-day BOD (BOD_5_), may not necessarily exert the ultimate biochemical oxygen demand because 5 days may be too short a time for complete stabilization by the bacteria. Thus we can summarize

$$\begin{array}{l} \text{BOD ultimate}=\text{Energy Oxygen}+\text{Endogenous Oxygen}\\ {\text{BOD}}_{5}\;\text{may be some percentage of BOD ultimate} \end{array}$$

*Energy Oxygen*, EO, is that amount of oxygen needed in energy reactions to provide synthesis of new biological cells and/or biologically stable organic substances from organic matter (bacterial food or organic pollutant) until none of the organic matter remains. This process is relatively rapid compared to endogenous respiration (consumption of endogenous oxygen) [3,4].

*Endogenous Oxygen* is that amount of oxygen used in reducing cellular materials to stable end products by a cyclic degradation process consisting of cell lysis—cell synthesis—cell lysis. Thus, a mass of bacterial cells is reduced ultimately to a relatively stable mass where life is maintained. This is a relatively slow process whose rate and extent of bacterial colony size is dependent on temperature. Thus the standard BOD_5_ is determined at a given temperature, 20 °C, to obtain reproducible results.

The ABODA instrument, an acronym for automated biochemical oxygen demand analyzer, consists of an analyzing system and subsidiary systems which supply a measured volume of sample, standardize the dissolved oxygen probe and recorder scale, etc. The analyzer system is a loop containing an aeration cell in which the sample diluted with water is saturated with air, a pump, a Biological Reactor, a dissolved oxygen (DO) probe and finally, the aeration cell. The diluted sample is pumped around this loop, and the saturated DO level achieved in the aeration cell is decreased due to the interaction of the microorganisms in the Biological Reactor, the DO and the pollutant sample (food). The decreased DO level is measured by the DO probe. The signal from the DO probe is traced out on a recorder. An electronic integrator gives the area under the curve which represents the amount of oxygen consumed by the microorganisms in response to the sample [5,8]. This value is proportional to the energy oxygen. The energy oxygen is calculated using several factors such as a calibration factor for the electrode, scaling factor for the integrator, flow rate in the analysis system and sample size.

The EO diagram is obtained from the ABODA instrument, and the EO is obtained by integrating the area under the curve and calculating as follows:

$$\text{EO}=\left({\text{Area, mg of}\;0}_{2}/L\right)\times \left(\text{time},\;t_{f}-t_{i},\min\right)\times \left(\text{flow rate, mL/min}\right)/\left(\text{volume of sample, mL}\right)$$

The correlation of EO and BOD_5_ values for five different concentrations of glucose—glutamate standards is given in table 1. A regression analysis resulted in a correlation coefficient, *r*^2^, of 0.99.

A series of raw sewage and primary (1°) effluent samples obtained from two different waste treatment plants were analyzed for their EO and BOD_5_ values (see table 2). Plant R is a small domestic treatment plant with flow rates from 3 to 5 MGD, while plant BC treats a mixture of domestic and industrial wastes with a flow rate of about 100 MGD. A correlation coefficient, *r*^2^, of 0.92 was obtained in a regression analysis of these data.

A number of secondary effluent samples from three different plants were analyzed for their EO and BOD_5_ values. Some of these values fall on a “best fit” line. However more extensive testing is needed to allow regression analysis for secondary effluent samples.

In all the EO determinations carried out in this investigation, the analysis time was an hour or less. The overall precision of EO analyses by the ABODA instrument and BOD_5_ is given as weighted average % relative standard deviation value in table 3.

In summary, the ABODA instrument measures EO which is correlated with BOD_5_, precise (±5.4% SD), and it accomplishes an analysis in 1 hour.

## Figures and Tables

**Table 1:** Correlation of EO and BOD, for glucose-glutamate

BOD_5_ mg/L	EO mg/L
217	63.5, 57.9, 63.9
160	41.6
108	31.6
58.6	12.3, 15
27	7.8, 6.3

**Table 2:** Correlation studies of waste samples

Plant	Sample	EO	BOD_5_
R	Raw	4.49	50.5
BC	Raw	5.91	61.0
BC	Raw	4.52	49.8
R	Raw	6.11	75.3
R	1°	3.21	34.1
BC	1°	5.90	39.8

**Table 3:** Weighted average of 1% rel. std. dev. data

Sample	EO			BOD_5_
	%Rel. s.d.	No. analyses	%Rel. s.d.	No. analyses
Glucose-glutamate	±4.3	67	±4.4	29
Waste samples	±6.1	106	±9.6	111
Weighted avg.	±5.4	173	±8.5	140
